# Supplementary material for: Scalable Fabrication of Thermally Conductive Layered Nacre-like Self-Assembled 3D BN-Based PVA Aerogel Framework Nanocomposites
Source: Polymers (Basel). 2022 Aug 15;14(16):3316. doi: 10.3390/polym14163316 (PMC9412551; doi:10.3390/polym14163316)
Supplement: Supplementary file 1 [file polymers-14-03316-s001.zip › polymers-1854493-supplementary.pdf]

Supplementary Information

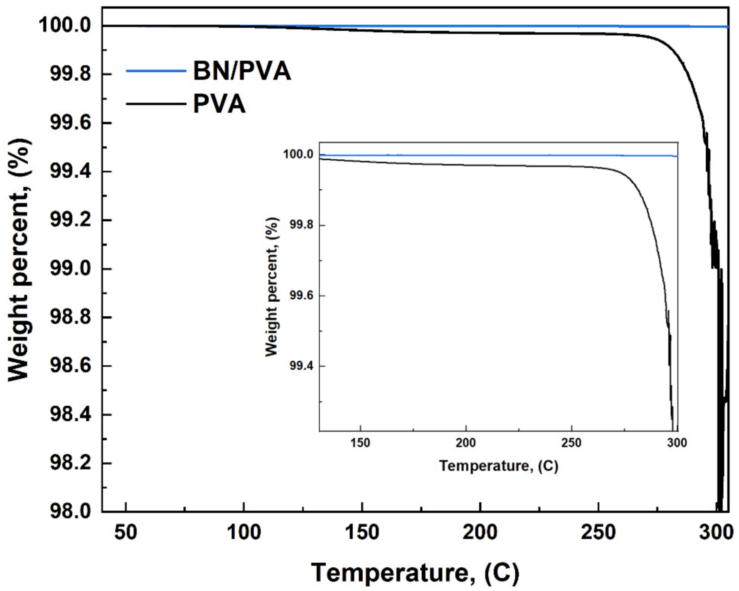

Figure S1. *TGA curves of PVA and BN/PVA aerogel nanocomposites*

BN/PVA aerogel cake frameworks show an outstanding thermal stability at even 800°C with no degradation of temperature while neat PVA showed full degradation of material at 300°C. Even for comparison at 1% degradation temperature, BN/PVA composite doesn't degrade at all while neat PVA reached the temperature of 295°C.

Table S1. *Data derived from TGA curves of nanocomposites at 1% degradation temperature.*

Filler content, (wt%)	$T_{1\%}$ (ºC)
Neat PVA	295
BN/PVA	Doesn't degrade

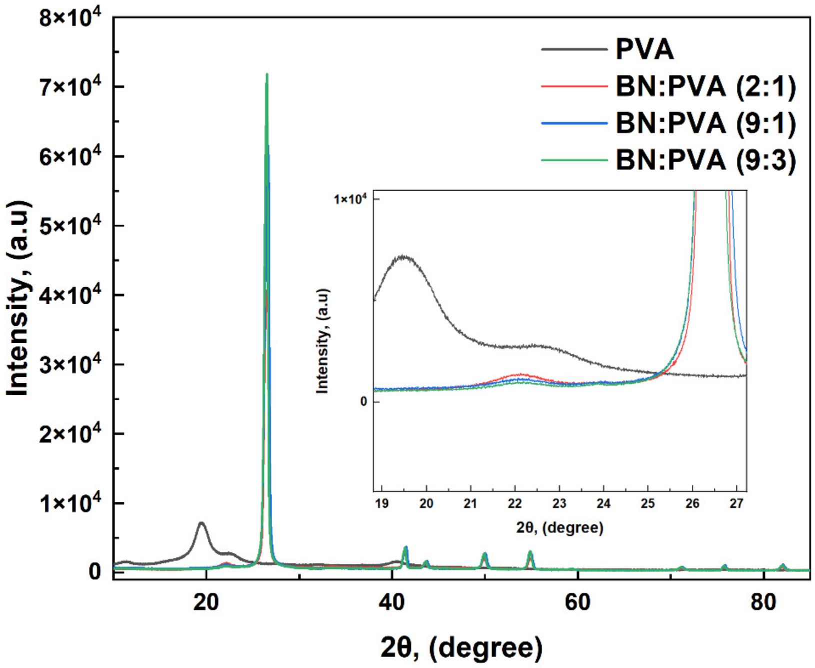

Figure S2. *XRD pattern of PVA and BN/PVA composites.*

The XRD pattern of as-prepared BN based PVA composites shows several distinctive diffraction peaks at 22.3°, 41.5°,43.6°, 50.1°,54.6°, 71.1°, and 82.6°. Except for the characteristic diffraction peaks of neat PVA, no obvious deviation or difference in diffraction peaks was found for any of the BN/PVA composite aerogels when compared to the neat PVA. Among all, some diffraction peaks at 22.3° and 41.5° exhibit the grafting of PVA in BN to some extent.

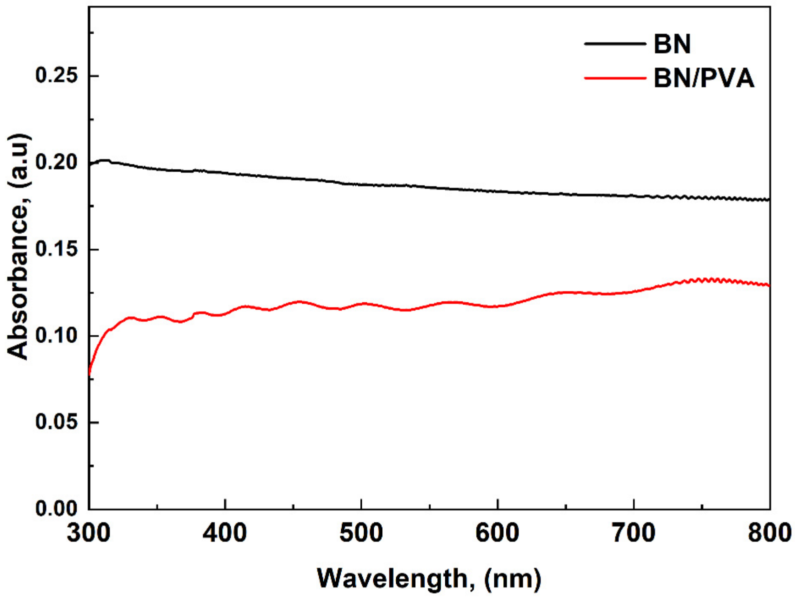

Figure S3. *UV-Vis absorption spectra of PVA and BN/PVA (9:1) composite aerogels.*

The UV-Vis absorption spectra of PVA and BN/PVA composite aerogel samples are shown in the Figure S3. The BN/PVA aerogel cake exhibits transparency due to its low absorption intensity across a broad range of wavelengths. Due to limited light absorption in the UV region, pure PVA had weak UV blocking properties then pure BN. The ability to absorb UV radiation was far better in pure BN compared to BN/PVA hybrid composite. As the BN concentration to PVA in the aerogel changed from (1:0) to (9:1), the intensity of UV absorption of aerogel decreased noticeably. The outcomes show that PVA gave BNNSs aerogels significant UV blocking properties.

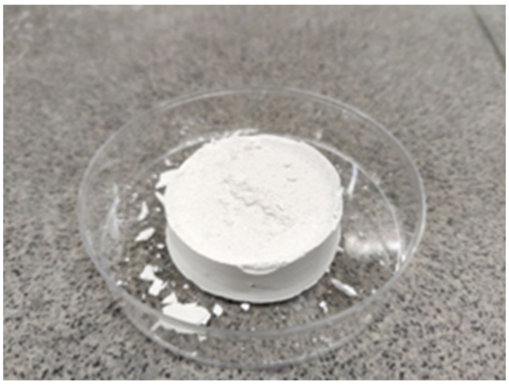

Figure S4. *Typical BN/PVA aerogel prototype with the stoichiometric ratio of (2:1).*

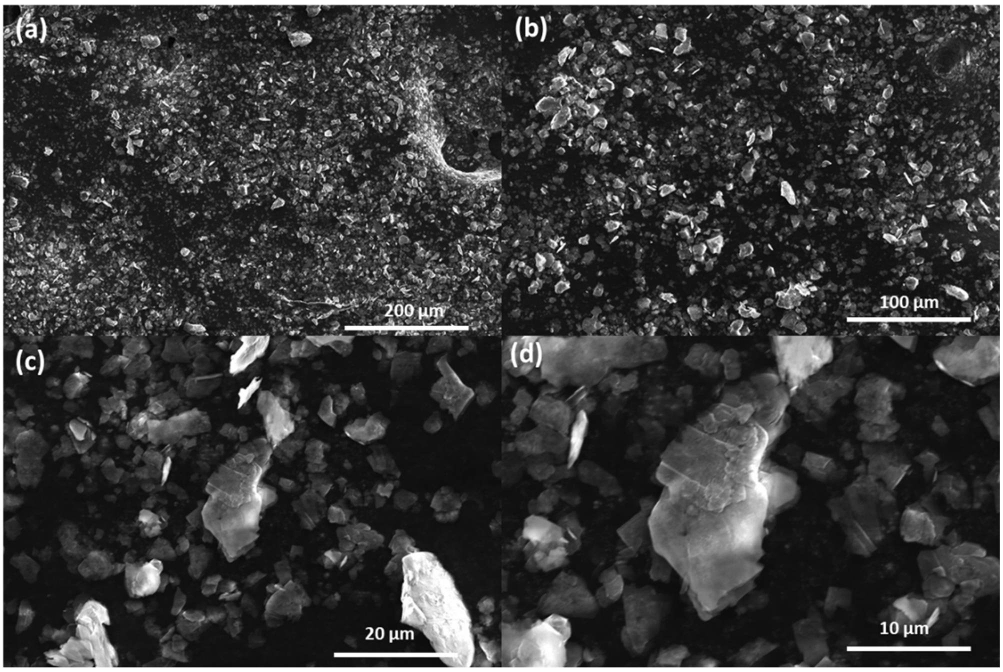

Figure S5. SEM images of pristine BN with an average particle size of ~ 10µm at different magnifications.
